# Green Nanoparticles for Mosquito Control

**DOI:** 10.1155/2014/496362

**Published:** 2014-08-27

**Authors:** Namita Soni, Soam Prakash

**Affiliations:** Advance and Environmental Parasitology, Vector Control Biotechnology and Biomedical Laboratories, Department of Zoology, Faculty of Science, Dayalbagh Educational Institute, Dayalbagh, Agra 282 110, India

## Abstract

Here, we have used the green method for synthesis of silver and gold nanoparticles. In the present study the silver (Ag) and gold (Au) nanoparticles (NPs) were synthesized by using the aqueous bark extract of Indian spice dalchini (*Cinnamomum zeylanicum*) (*C. zyelanicum* or *C. verum* J. Presl). Additionally, we have used these synthesized nanoparticles for mosquito control. The larvicidal activity has been tested against the malaria vector *Anopheles stephensi* and filariasis vector *Culex quinquefasciatus*. The results were obtained using UV-visible spectrophotometer and the images were recorded with a transmission electron microscope (TEM). The efficacy tests were then performed at different concentrations and varying numbers of hours by probit analysis. The synthesized AgNPs were in spherical shape and average sizes (11.77 nm AgNPs and 46.48 nm AuNPs). The larvae of *An. stephensi* were found highly susceptible to the synthesized AgNPs and AuNPs than the *Cx. quinquefasciatus*. These results suggest that the *C. zeylanicum* synthesized silver and gold nanoparticles have the potential to be used as an ideal ecofriendly approach for the control of mosquito.

## 1. Introduction

Cinnamon is a small evergreen tree, which belongs to family Lauraceae. It is the native of Sri Lanka and Southern India. It has antioxidant, antimicrobials, mosquito control, and other properties [1]. Mosquitoes are vectors of many diseases, including malaria, filariasis, dengue, and Japanese encephalitis. Among these kinds of malaria, spread by the bite of female* Anopheles* mosquito, and filariasis, spread by* Culex* mosquito, are the two vector borne diseases of the tropical region and are considered major public health concerns.

According to WHO, there were about 219 million cases of malaria in 2010 (with an uncertainty range of 154 million to 289 million) and an estimated 660,000 deaths (with an uncertainty range of 490,000 to 836,000). Malaria mortality rate has fallen by more than 25% globally since 2000 and by 33% in the WHO African region. Most deaths occur among children living in Africa, where malaria claims the life of a child every minute. Country-level burden estimates available for 2010 show that an estimated 80% of malaria deaths occur in just 14 countries and about 80% of cases occur in 17 countries. Together, the Democratic Republic of the Congo and Nigeria account for over 40% of the total estimated malaria deaths globally [2].

On the other hand, nearly, 1.4 billion people in 73 countries worldwide are threatened by lymphatic filariasis, commonly known as elephantiasis. Over 120 million people are currently infected, with about 40 million disfigured and incapacitated by the disease [3]. Control or eradication of the mosquito population could significantly restrict the spread of disease.

Synthesizing nanoparticles using plants and microorganisms can eliminate this problem by making the nanoparticles more biocompatible. The synthesis of silver nanoparticles from silver precursor using the bark extract and powder of novel* Cinnamon zeylanicum* has been reported [4]. The potential of nanocrystalline palladium particle production using* Cinnamomum zeylanicum *(*C. zyelanicum* or* C. verum* J. Presl) bark extract has been studied [5].

The silver and gold nanoparticles using* A. calamus* [6],* E. sativa* and* S. oleracea* [7],* T. conoides* [8], and* A. nilotica* [9] have been synthesized.

The larvicidal activity of biosynthesized silver nanoparticles using the leaf extracts of* P. pinnata* [10] and* L. aspera* [11] has been tested against the dengue vector* Ae. albopictus* and* Ae. aegypti*.

In the present investigation we have synthesized the AgNPs and AuNPs nanoparticles by using the bark extract of* C. zeylanicum*. Further, the synthesized nanoparticles have also been tested against the larvae of* Anopheles stephensi* and* Culex quinquefasciatus*. The* C. zeylanicum* synthesized AgNPs and AuNPs have the potential to be used as an ideal ecofriendly approach for the control of mosquito.

## 2. Materials and Methods

### 2.1. Material

The bark of* Cinnamomum zeylanicum* (*C. zyelanicum* or* C. verum* J. Presl) was collected from the local market of Agra, India. The voucher specimen is maintained in our laboratory for future use.

### 2.2. Extract Preparation

The bark of* Cinnamomum zeylanicum* (*C. zyelanicum* or* C. verum* J. Presl) was washed with distilled water for removing the dust particles. The bark was air-dried and converted into powder and a bark broth was prepared by placing 10 g of bark powder in a 250 mL of deionized water. This mixture was boiled at 60°C, for 5 min, decanted, or filtered through Whatman-1 filter paper.

### 2.3. Nanoparticles Synthesis

After obtaining the aqueous extract of bark, the filtrates were treated with aqueous 1 mM AgNO_3_ and HAuCl_4_ solutions in an Erlenmeyer flask and incubated at room temperature. Formation of AgNPs and AuNPs was indicated by the dark brown and purple coloration of the solutions.

### 2.4. Characterization of Nanoparticles

Synthesis of AgNPs and AuNPs was confirmed by sampling the reaction mixture at regular intervals, and the absorption maxima were scanned by UV-Vis spectra, at the wavelength of 350–750 nm in a UV-3600 Shimadzu spectrophotometer at 1 nm resolution. The micrographs of AgNPs and AuNPs were obtained by TECHNAI 200 Kv TEM (Fei, Electron Optics) transmission electron microscope. For transmission electron microscopy analysis, samples were prepared on carbon coated copper TEM grids.

### 2.5. Rearing of Mosquito Larvae

The larvae of* Cx. quinquefasciatus* and* An. stephensi* were collected from various localities including urban, rural, and semiurban regions of Agra (27°, 10′N, 78°05′E), India. The larvae were reared in deionized water containing glucose and yeast powder. The colonies of* Cx. quinquefasciatus* and* An. stephensi* were maintained in the laboratory at a temperature of 25°C with a relative humidity of 75 ± 5% and 14 h of photoperiod. The larvae of* Cx. quinquefasciatus* and* An. stephensi* were maintained in separate enamel containers as per the standard method [12].

### 2.6. Bioassays, Data Management, and Statistical Analysis

AgNPs and AuNPs synthesized from* Cinnamomum zeylanicum* (*C. zyelanicum* or* C. verum* J. Presl) were tested for their killing activities against the larvae of* Cx. quinquefasciatus* and* An. stephensi* and were assessed by using the standard method [13]. All larvae of* Cx. quinquefasciatus* and* An. stephensi* were separated and placed in a container in microbe-free deionized water. After that, different test concentrations of AgNPs and AuNPs in 100 mL deionized water were prepared in 250 mL beakers. Bioassays were conducted separately for each instar at five different concentrations of aqueous AgNPs and AuNPs (2, 4, 6, 8, 10 ppm). To test the larvicidal activity of our AgNPs and AuNPs, 20 larvae of each stage were separately exposed to 100 mL of test concentrations. Thereafter, we examined their mortality after different time of treatment during the experimental periods.

The data on the efficacy were subjected to probit analysis [14]. The control mortality was corrected by Abbott's formula [15].

## 3. Results

### 3.1. Analysis of UV-Vis Spectra

**Figure 1:**
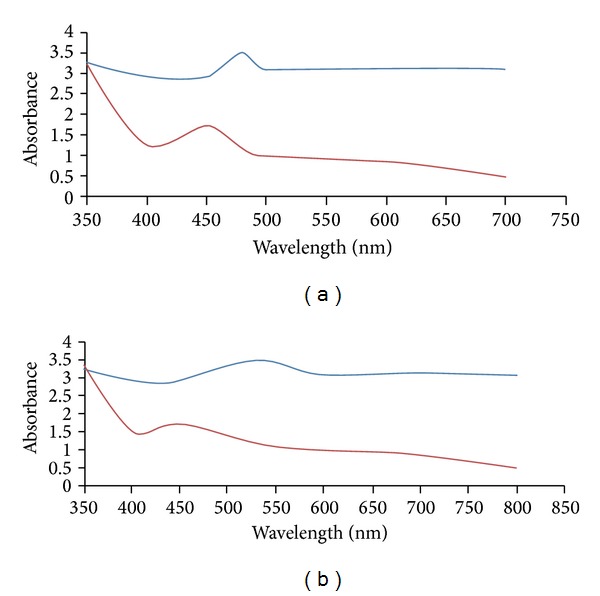
UV-Vis spectra of (a) silver nanoparticles and (b) gold nanoparticles synthesized by using the bark extract of* Cinnamomum zeylanicum* recorded from reaction medium before (1) and after immersion of AgNO_3_ (2) after 24 h.

The aqueous extracts of bark of* C. zeylanicum *(*C. zyelanicum* or* C. verum* J. Presl) were light yellow and brown in colour before immersion in AgNO_3_ and HAuCl_4_ solutions. The colour of bark aqueous extract changed into dark red and purple colour after immersing in AgNO_3_ and HAuCl_4_ solutions after 72 h of incubation. The change in colour is a signal for the formation of AgNPs and AuNPs. Figures 1(a) and 1(b) show the UV-Vis spectra of synthesized AgNPs and AuNPs using the bark of* C. zeylanicum* (*C. zyelanicum* or* C. verum* J. Presl) recorded from reaction medium before (1) and after immersion of AgNO_3_ and HAuCl_4_ (2) after 72 h. Absorption spectra of AgNPs and AuNPs formed in the reaction medium have a broad absorption band centred at 480 nm and 530 nm c.a.

### 3.2. TEM Analysis

**Figure 2:**
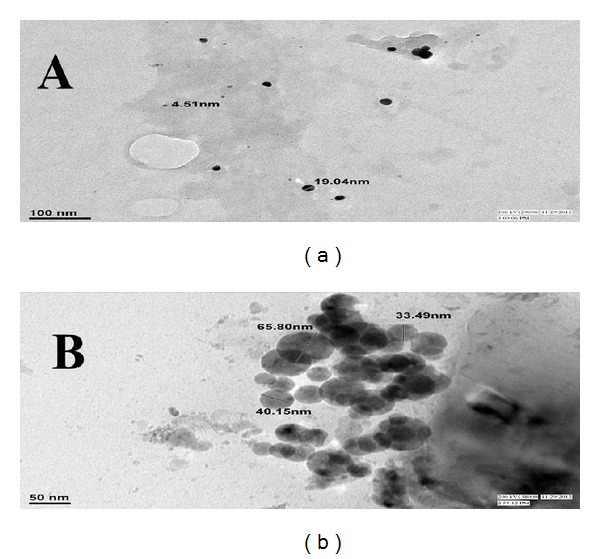
TEM micrographs of* Cinnamomum zeylanicum* synthesized (a) silver nanoparticles and (b) gold nanoparticles.

Figures 2(a) and 2(b) show the TEM micrographs of synthesized AgNPs and AuNPs. The average size of AgNPs is 11.77 nm and AuNPs 46.48 nm and they were spherical-shaped.

### 3.3. Efficacy of Synthesized AgNPs and AuNPs against* An. stephensi* Larvae

The efficacy of the synthesized AgNPs and AuNPs was tested against the larvae of* An. stephensi*. Different concentrations of aqueous AgNPs and AuNPs (2, 4, 6, 8, 10 ppm) were tested against the larvae of* An. stephensi*.

The larvae of* An. stephensi* were found highly susceptible to the synthesized AgNPs. First instar larvae (LC_50_ 2, LC_90_ 11, and LC_99_ 12 ppm), second instar larvae (LC_50_ 10, LC_90_ 15, and LC_99_ 17 ppm) after 4 h, third instar larvae (LC_50_ 6, LC_90_ 11, and LC_99_ 13 ppm), and fourth instar larvae (LC_50_ 10, LC_90_ 15, and LC_99_ 17 ppm) after 22 h of exposure were obtained with their confidence limits and *χ* ^2^ and *r* values (Table 1).

**Figure 3:**
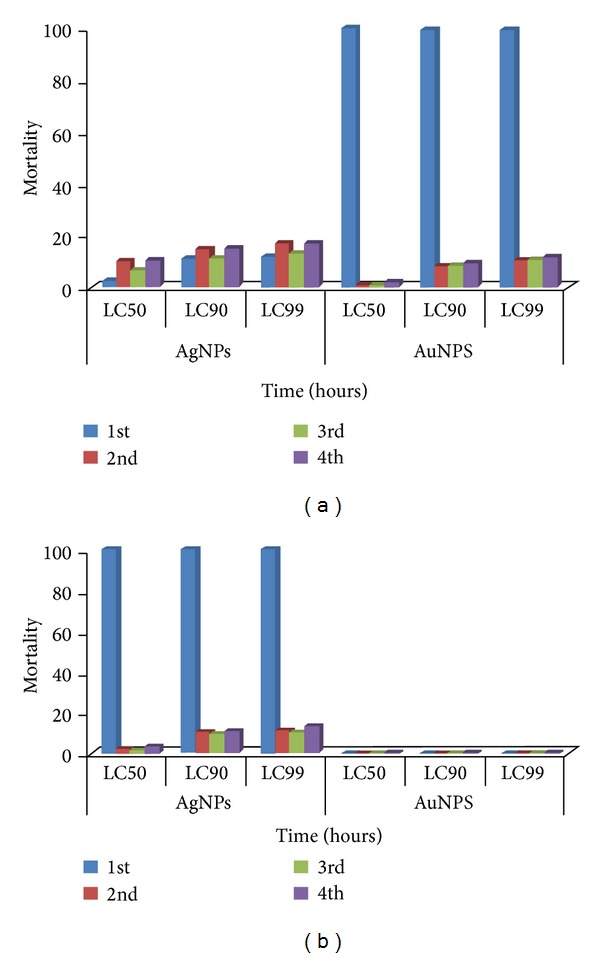
Efficacy of silver and gold nanoparticles against the larvae of* Anopheles stephensi* at different hours of exposure.

The larvae of* An. stephensi* were found susceptible to the synthesized AuNPs. The first instar larvae have shown the 100% mortality after 24 h of exposure. The second instar larvae (LC_50_ 1, LC_90_ 8, and LC_99_ 10.5 ppm) after 24 h, third instar larvae (LC_50_ 1, LC_90_ 8, and LC_99_ 10.5 ppm), and fourth instar larvae (LC_50_ 2, LC_90_ 10, and LC_99_ 11 ppm) after 72 h of exposure were obtained with their confidence limits and *χ* ^2^ and *r* values (Table 1, Figure 3(a)).

### 3.4. Efficacy of Synthesized AgNPs and AuNPs against* Cx. quinquefasciatus* Larvae

The efficacy of the synthesized AgNPs and AuNPs was tested against the larvae of* Cx*.* quinquefasciatus*. Different concentrations of aqueous AgNPs and AuNPs (2, 4, 6, 8, 10 ppm) were tested against the larvae of* Cx*.* quinquefasciatus*.

The larvae of* Cx*.* quinquefasciatus* were found highly susceptible to the synthesized AgNPs. The first instar larvae have shown the 100% mortality after 24 h of exposure. The second instar larvae (LC_50_ 2, LC_90_ 10, and LC_99_ 11 ppm), third instar larvae (LC_50_ 1.5, LC_90_ 9, and LC_99_ 10.5 ppm), and fourth instar larvae (LC_50_ 4, LC_90_ 11, and LC_99_ 13 ppm) after 24 h of exposure were obtained with their confidence limits and *χ* ^2^ and *r* values (Table 2).

The larvae of* Cx*.* quinquefasciatus* were found less susceptible to the synthesized AuNPs. No mortality was observed after 24 h of exposure (Table 2, Figure 3(b)).

## 4. Discussion

In the present investigation we have synthesized the AgNPs and AuNPs by using the bark of* C. zeylanicum* (*C. zyelanicum* or* C. verum* J. Presl). The efficacy of synthesized NPs has been tested against the larvae of malaria vector* An. stephensi* and filariasis vector* Cx. quinquefasciatus*.

An economically viable and “green chemistry” approach for biological synthesis of silver nanoparticles using aqueous leaf extract of* P. dulce* has been reported as larvicidal activity against the* Cx. quinquefasciatus* previously [16]. The larvicidal activity of biogenic nanoparticles against filariasis causing* Culex* mosquito vector has also been evaluated before [17].

Larvicidal activity of silver nanoparticles (AgNPs) using leaf extract of* N. oleander* (Apocynaceae) against the first to the fourth instar larvae and pupae of malaria vector,* An. stephensi* (Diptera: Culicidae), was carried out in an earlier study [18]. The fabrication, characterization, and mosquito larvicidal bioassay of silver nanoparticles synthesized from aqueous fruit extract of* Putranjiva* and* D. roxburghii* were observed [19]. Moreover, the activity of silver nanoparticles (AgNPs) synthesized using* M. koenigii* plant leaf extract against the first to the fourth instars larvae and pupae of* An. stephensi* and* Ae. aegypti* was determined, too [20]. Among these previous studies, the silver nanoparticles were synthesized by using the aqueous extract of leaf and other parts of plant extract and nanoparticles were tested against the first and the fourth instar larvae and pupae of mosquitoes. However, in the present study we have synthesized silver and gold nanoparticles by using aqueous extracts of bark of* C. zeylanicum *(*C. zyelanicum* or* C. verum* J. Presl). These nanoparticles were tested as larvicide against* An. stephensi* and* Cx. quinquefasciatus*.

Recently, the larvicidal activity of silver nanoparticles synthesized by leaf extract of* P. pinnata* has been tested against the larvae of dengue vector* Ae. albopictus* [10]. Further, the larvicidal potential of silver nanoparticles synthesized from* L. aspera* leaf extract has been tested against the dengue vector* Ae. aegypti* [11]. However, the larvicidal activity of silver nanoparticles (AgNPs) synthesized using* F. elephantum* plant leaf extract against late third instar larvae of* An. stephensi*,* Ae. aegypti*, and* Cx. quinquefasciatus* has been determined [21]. Furthermore, activity of aqueous leaf extract and silver nanoparticles (AgNPs) synthesized using* H. indicum* plant leaves against late third instar larvae of* Ae. aegypti*,* An. Stephensi,* and* Cx. quinquefasciatus* has been studied [22]. Larvicidal activity of silver nanoparticles synthesized from aqueous leaf extract of* C. collinus* against the larvae of* Ae. aegypti* has now been determined [23], while in our study we have synthesized silver nanoparticles by using aqueous extracts of bark of* C. zeylanicum* (*C. zyelanicum* or* C. verum* J. Presl). These nanoparticles were tested as larvicide against* An. stephensi* and* Cx. quinquefasciatus*.

The larvicidal activities of synthesized cobalt nanoparticles using the biocontrol agent,* B. thuringiensis,* have been investigated against the malaria vector* An. subpictus* and dengue vector,* Ae. aegypti* [24]. Furthermore, the larvicidal activity of silver nanoparticles synthesized by using* B. thuringiensis* has been revealed against the* Ae. aegypti* [25], whereas in the present study we have tested the larvicidal activity of silver and gold nanoparticles synthesized by using aqueous extracts of bark of* C. zeylanicum *(*C. zyelanicum* or* C. verum* J. Presl). These nanoparticles were tested as larvicide against* An. stephensi* and* Cx. quinquefasciatus*.

Efficacy of fungus mediated silver and gold nanoparticles has been tested against the larvae of* An. stephensi, Cx. Quinquefasciatus,* and* Ae. aegypti* [26–28]. Furthermore, the larvicidal and pupicidal activities of silver and gold nanoparticles synthesized by fungi have also been investigated against* An. stephensi, Cx. Quinquefasciatus,* and* Ae. aegypti* [29–31]. Recently the silver nanoparticles have been synthesized by using the leaf and stem of* Piper nigrum* for their antibacterial activity against agriculture plant pathogens [32]. The silver nanoparticles have synthesized by using the leaf of* Pimenta dioica* [33]. However, in the present study the silver and gold nanoparticles have been synthesized by bark of* C. zeylanicum* against the larvae of* An. stephensi* and* Cx. quinquefasciatus*.

## 5. Conclusion

In this study we have synthesized the silver and gold nanoparticles by using the bark of* Cinnamomum zeylanicum *(*C. zyelanicum* or* C. verum* J. Presl). The larvicidal activity of the synthesized nanoparticles has been tested against the larvae of malaria vector* Anopheles stephensi* and filariasis vector* Culex quinquefasciatus*. The synthesized AgNPs were in spherical shape and average sizes (11.77 nm AgNPs, 46.48 nm AuNPs). The synthesized nanoparticles were found effective against the larvae of mosquito species. The results suggest that the* C. zeylanicum* (*C. zyelanicum* or* C. verum* J. Presl) synthesized silver and gold nanoparticles have the potential to be used as an ideal ecofriendly approach for the control of mosquito.

## Figures and Tables

**Table 1:** Efficacy of silver nanoparticles and gold nanoparticles synthesized by bark extract of *Cinnamomum zeylanicum* against the larvae of *Anopheles stephensi *with their LC values, 95% confidential limits (CL), and *χ* ^2^ and *r* values after different time of exposure.

NPs	Instar	Time	LC_50_ (95% CL)	LC_90_ (95% CL)	LC_99_ (95% CL)	*χ* ^2^	*r*
AgNPs	1st	4 h	2 (0.86–3.14)	11 (9.77–12.23)	12 (10.54–13.46)	50.29	0.82
	2nd	4 h	10 (8.86–11.14)	15 (13.77–16.23)	17 (15.54–18.46)	39.43	0.95
	3rd	22 h	6 (4.86–7.16)	11 (9.77–12.23)	13 (11.54–14.46)	43.83	0.95
	4th	22 h	10 (8.86–11.14)	15 (13.83–16.17)	17 (15.77–18.77)	37.66	0.89

AuNPs	1st	24 h	∗∗	∗∗	∗∗	∗∗	∗∗
	2nd	24 h	1 (0.86–2.14)	8 (6.83–9.17)	10.5 (9.27–11.73)	61.61	0.91
	3rd	72 h	1 (0.86–2.14)	8 (6.83–9.17)	10.5 (9.27–11.73)	61.61	0.91
	4th	72 h	2 (0.88–3.12)	10 (8.86–11.14)	11 (9.87–12.13)	54.93	0.89

∗∗100% mortality.

**Table 2:** Efficacy of silver nanoparticles and gold nanoparticles synthesized by bark extract of *Cinnamomum zeylanicum* against the larvae of *Culex quinquefasciatus *with their LC values, 95% confidential limits (CL), and *χ* ^2^ and *r* values after different time of exposure.

NPs	Instar	Time	LC_50_ (95% CL)	LC_90_ (95% CL)	LC_99_ (95% CL)	*χ* ^2^	*r*
AgNPs	1st	24 h	∗∗	∗∗	∗∗	∗∗	∗∗
	2nd	24 h	2 (0.88–3.12)	10 (8.86–11.14)	11 (9.87–12.13)	54.93	0.89
	3rd	24 h	1.5 (1.36–2.64)	9 (7.77–10.23)	10.5 (9.17–11.83)	58.85	0.85
	4th	24 h	4 (2.86–5.14)	11 (9.77–12.23)	13 (11.54–14.46)	49.41	0.87
AuNPs	Larvae	24 h	—	—	—	—	—

∗∗100% mortality.

—no mortality.
